# Life style, Perfusion deficits and Co-morbidities Precipitate Inflammation and Cerebrovascular Disorders in Aged Subjects

**DOI:** 10.15190/d.2015.31

**Published:** 2015-03-31

**Authors:** Adriana Uzoni, Ovidiu Ciobanu, Raluca Elena Sandu, Ana Maria Buga, Aurel Popa-Wagner

**Affiliations:** Department of Psychiatry, University of Medicine Rostock, Germany; Biochemistry Department, University of Medicine and Pharmacy "Victor Babes", Timisoara, Romania; Center of Clinical and Experimental Research, University of Medicine and Pharmacy, Craiova, Romania; Department of Psychiatry, University Medicine of Saarland, Homburg/Saar, Germany

**Keywords:** Inflammation, aging, depression, perfusion deficits, obesity, stroke

## Abstract

Cerebrovascular diseases represent 2nd leading cause of death worldwide. Understanding how genetic predispositions and their interaction with environmental factors affect cerebrovascular diseases is fundamental for prevention, diagnosis and for the development of safe and efficient therapies. Cerebrovascular diseases have not only a very high mortality rate, but also results in debilitating neurological impairments or permanent disability in survivors associated with huge economic losses. Among the women and men individuals with a low-risk lifestyle (smoking, exercising daily, consuming a prudent diet including moderate alcohol and having a healthy weight during mid-life) had a significantly lower risk of stroke than individuals without a low-risk lifestyle. Current review focuses on determining the relationship between diet, as an important component of ‘life style’, aging and cerebrovascular diseases.This review may help to unravel biological mechanisms linking lifestyle, diet-induced, metabolic inflammation, aging and cerebral hypoperfusion to development of cerebrovascular diseases, a prerequisite for development of science-based preventive strategies needed to combat the major public health challenges like obesity and stroke.

## 1. Introduction: the global burden of cerebrovascular diseasesroduction: the global burden of cerebrovascular diseases

Cerebrovascular diseases are among the most prevalent health care problems in Europe. Total European cost of brain disorders in 2010 was 798 billion € of which 64.1 billion € was related to stroke alone^1^. In many cases, the result of cerebrovascular disorders is a loss of independent living and secondary health problems affecting not only patients but also their families. The number of elderly people is increasing with a number of co-morbidities increasing the risk of cerebrovascular diseases (CVD)^2^. Thus, strategies in guiding patient selection and novel preventive and neuroprotective therapies are urgently needed. Emerging evidence suggest that several diseases show overlapping pathology with neuroinflammation as one possible common mechanism leading to increased risk of cerebrovascular disorders.

In demographically developed countries, the average age at which stroke occurs is around 73 years reflecting the older age structure of these countries. The probability of a first stroke or first transitory ischemic attack is around 1.6 per 1,000 and 0.42 per 1,000, respectively. In less developed regions, the average age of stroke will be younger due to the different population age structure resulting from higher mortality rates and competing causes of death (The World Health Report, 2002).

Stroke patients are at highest risk of death in the first weeks after the event, and between 20% to 50% die within the first month depending on type, severity, age, co- morbidity and effectiveness of treatment of complications. Patients who survive may be left with no disability or with mild, moderate or severe disability. Considerable spontaneous recovery occurs up to about six months^3^. However, patients with a history of stroke are at risk of a subsequent event of around 10% in the first year and 5% per year there after^4^.

The negative consequences of stroke extend well beyond the victims themselves, ultimately including families, caregivers, social networks and employers. The proportion of patients achieving independence in self-care by one year after a stroke ranges from around 60% to 83%. This wide variation relates to whether the studies are community based or hospital based, which activities are considered in estimating independence, and the methods used to rate ability. In established marked economies (EMEs), depending on the organization of hospital services, between 10% and 15% of survivors are resident in an institution at one year^5^.

Worldwide stroke incidence is increasing in parallel with modernization, changes in lifestyle, and the growing elderly population. In particular, rates in Eastern Europe have been increasing, such that currently the highest rates are found in countries such as Bulgaria, Romania, and Hungary. Among the women and men individuals with a low-risk lifestyle (no smoking, exercising daily, consuming a prudent diet including moderate alcohol and having a healthy weight during mid-life) had a significantly lower risk of stroke than individuals without a low-risk lifestyle. Therefore the relatively high incidence of stroke may be due in part to the impact of numerous known risk factors in these populations^6^: arterial hypertension, diabetes, high cholesterol, smoking, alcoholism, obesity, stress, and a sedentary lifestyle.

New methods of diagnosis and treatment have improved the management of known risk factors (such as hypercholesterolemia and high blood pressure) as well as the sequelae of stroke, such that decreases both in stroke incidence (by 20% in the 1980’s), and in mortality induced by stroke (by about 3-5% per year in Europe between the years 1970 and 1985) have been recorded^7^. At the same time, however, those vulnerable to stroke will become more numerous as the elderly population rapidly increases around the world, and many risk factors remain either unknown or beyond the reach of current therapies (as in the case of old age, the most important, and growing, risk factor for stroke).

## 2. Mechanisms linking lifestyle, diet-induced, metabolic inflammation to development of cerebrovascular diseases

Although CVDs to some extent are predictable through anthropometric, lifestyle, and clinical factors, the metabolic pathways underlying the progression of disease are only poorly understood.

Fuelled by rapid urbanization, westernised diet and an increasingly sedentary lifestyle, the Type2 Diabetes Mellitus epidemic has grown in parallel with the worldwide rise in obesity. For several decades, the diet-heart paradigm that high intake of saturated fat and cholesterol increases the risk of atherosclerosis and CVDs has been the driving force behind national and international dietary recommendations for prevention of CVD^8^.

Bad nutritional habits can lead to metabolic disorders, triggered by a system-wide chronic inflammation, also called metaflammation, metabolic inflammation^9^. A metaflammation state can lead to a series of disorders and diseases, including hypertension, metabolic syndrome, CVD, stroke, insulin resistance and type 2 diabetes mellitus (T2DM). It is postulated that lipid hormones including sphingolipids and eicosanoids in concert with cytokines and adipokines play an important role in this process by inducing adverse regulatory responses in target cells such as macrophages. The role of genetics in driving metabolic disease development is strongly indicated by the higher concordance rate of T2DM in monozygotic than in dizygotic twins. It has been estimated that 30% to 70% of T2DM risk can be attributed to genetics^10^. The investigation of gene-environment interactions through large collaborative efforts holds promise in furthering our understanding of the interplay between genetic and environmental factors^11^.

Since the completion of the HapMap project and the availability of whole genome single nucleotide polymorphisms (SNP) assays, genome-wide analysis of correlations between genetic variants and phenotypes has become an important approach to find disease-causative genes. Genome wide SNP typing is often performed in very large groups of human individuals (cohorts), and a large number of loci underlying disease have now been catalogued^12^ including variants that increase susceptibility to T2DM. However, these loci confer effects of only modest size and do not add to the clinical prediction of diabetes beyond that of traditional risk factors, such as obesity, physical inactivity, family history of diabetes, and certain clinical parameter. Furthermore, our contribution to identifying the genomic SNP pattern providing insights to biological basis of obesity^13^ and the identification of new genetic loci linking adipocyte and insulin biology to body fat distribution^14^, will enable differentiation between genetics and lifestyle factors. The combination of genome wide- association studies (GWAS) with metabolomics is now breaking new grounds^15^, as it allows making associations between SNPs and so-called intermediate phenotypes that can be obtained through exact measurements. Metabolomics facilitates the exact quantitative measurement of large sets of lipid molecules and other metabolites, and GWAS has allowed the mapping of numerous metabolic phenotypes on the genome, as demonstrated by the discovery of substantial numbers of loci with relative strong effects^16-19^.

## 3. Obesity, inflammation and rehabilitation odds in stroke patients

The “obesity paradox” has been reported in many articles as a inverse relationship between the body mass index (BMI) and mortality in stroke patients. The relationship between BMI and functional recovery in post stroke patients has not been well described^20-25^.

In a cohort study from the China National Stroke Registry war analysed the relationship between the body mass index (BMI) , mortality and post stroke functional recovery at 3 months after disease onset. This study enrolled and analyzed 10,905 patients with eligible acute ischemic stroke. Favourable functional recovery was seen in 52,4 % of underweight (BMI 18,5 kg/m2) , 55,0% of normal weight (BMI 18,5-22,9 kg/m2), 61% of overweight (BMI 23-27,4 kg/m2) , 59,2% of obese (27,5-32,4 km/m2) and 60,3% of severe obese (BMI > 32,5 kg/m2) stroke survivors. The overweight AIS survivors had better 3-month functional recovery and obesity not severe obesity showed a protective trend. Second, severe obesity was associated with higher mortality and overweight/obesity was not a protective factor of survival at 3 months after stroke^26^.

In a study on the effect of BMI on stroke rehabilitation that included 819 patients admitted to an acute rehabilitation hospital for stroke rehabilitation, overweighted patients had, paradoxically, better functional progression than did patients in other weight categories^27^.

In still another study on the “Obesity Paradox” that included 510 with transient ischemic attack survivors showed that the excess of adiposity increases the chance of severe disability after ischemic stroke. Since BMI reflects also total lean mass, it is risky to conclude that there is a protective effect of obesity alone in the functional recovery after stroke. Nevertheless, it is possible that a certain magnitude of body mass is necessary to prevent severe disability in stroke survivors^28^.

Contradictory results were reported in a study from 2007 that showed that the functional improvement in normal weighted patients was better than that in overweight/obese patients^29^.

In a large retrospective cohort study from the Danish Stroke Register, 53812 patients were analyzed for BMI, age, sex, civil status, stroke severity, stroke subtype, a predefined cardiovascular profile, and the socioeconomic status. There was no evidence of an “obesity paradox” in patients with reported stroke. However, stroke occurred at a significantly younger age in patients with higher BMI.

Another study analyzed 451 patients hospitalized for ischemic stroke and found that BMI on admission had no relationship to functional recovery on discharge^30^. The association between higher BMI and favorable functional recovery might be influenced by stroke severity because patients with high BMI seemed to have had less severe strokes^25^ and more lacunar infarctions^31^.

## 4. Perfusion deficits in the elderly, inflammation and depression 

Recent work suggest that perfusion deficits in the elderly can trigger microglial activation and subsequent neuroinflammation^32,33^ ultimately resulting in demyelination and neurodegeneration^34^.

Previous studies in rodents indicate that aging and preclinical neurodegenerative disease processes promote proinflammatory states in older individuals and leads to the development of a primed and immune-reactive population of microglia^35-41^. Further, immune activation can be a characteristic of depression^42,43^ and precipitate depressive symptoms^44,45^. Moreover, recent work suggest that perfusion deficits in the elderly can trigger microglial activation and subsequent neuroinflammation and shifts the central nervous system (CNS) into a proinflammatory state^32,33,41^ ultimately resulting in demyelination and neurodegeneration^34^. This was particularly evident in middle aged rodents as compared to the young counterparts^46^.

Recent research suggests that the inflammatory process is potentially intricately linked with multiple neurodegenerative pathways for depression and pro-inflammatory cytokines^47^ and plays a central role in the pathophysiology of both depression and dementia^48-51^. There is strong evidence that in humans vascular disease vascular Abeta deposition in the brain promotes development of depression and increases the risk of dementia by causing loss of vascular autoregulation associated with rigidity of arterioles, leading to infarction in the territory of their branching vessels in the temporal cortex of patients with cerebral angiopathy (CAA). This is associated with marked vascular/perivascular infiltration of inflammatory cells, a condition mimicked in mice subjected to chronic cerebral hypoperfusion^52,53^.

## 5. Life style, co-morbidities and depression

It is becoming well established that lifestyle, especially dietary habits, greatly affect metabolic health. This model which promotes diets that are typically low in fat and high in carbohydrates (the ‘Western’ diet), has led to a substantial decline in the percentage of energy intake from total and saturated fats. At the same time, it has spurred a compensatory increase in consumption of refined carbohydrates and added sugars, a dietary shift that may be contributing to the current twin epidemics of obesity and diabetes: both metabolic diseases and identified as risk factors for CVD. The changed landscape in obesity and dietary patterns suggests a need to reassess the strategy of replacing total and saturated fats with carbohydrates.

Diabetes mellitus and depression and are two of the most common illnesses in the elderly. Both depression and diabetes increase reciprocally the risk of dementia. Recent reviews suggest that approximately 20% of adult patients with T2DM meet criteria for comorbid major depression. A systematically review aimed to estimate the prevalence of depression in adults with T2DM found that a personal history of depression are more likely to develop dementia and cognitive decline later in life. Recently, in a prospective study in patients with type 2 diabetes, 20% of the patients with diabetes met criteria for depression. Furthermore, patients with comorbid depression had a 100% increased risk of developing dementia during the 3 to 5 years study period^54-58^.

## CONCLUSIONS:

♦ Worldwide cerebrovascular diseases are increasing in parallel with modernization, changes in lifestyle, and the growing elderly population.

♦ Understanding how aging and life style precipitate the development of cerebrovascular diseases is fundamental for prevention, diagnosis and for the development of safe and efficient therapies.

♦ Genetic findings, once unambiguously identified, have the potential to predict an individual’s disease risk, phenotype, and possibly progression. They also may contribute to clinical management by prompting heightened surveillance, providing indication for preventive intervention, and guiding treatment selection.

**Figure 1:**
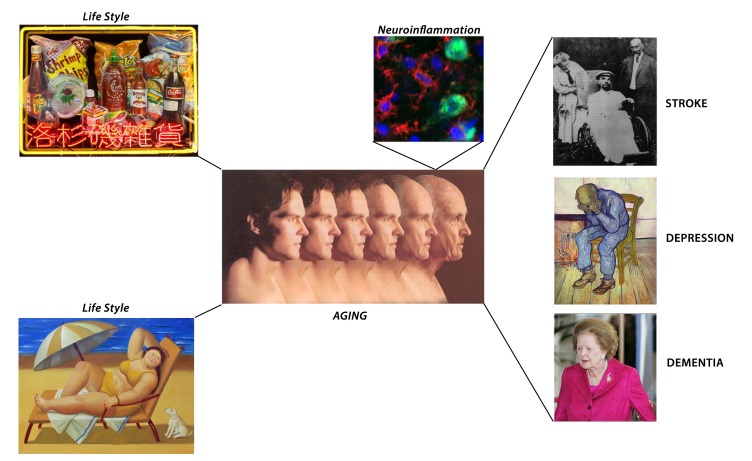
Life style, aging, hypoperfusion and neuroinflammation precipitate neuropsychiatric diseases as shown in art images Brain neuroinflammation is illustrated by Iba1 immunoreactivity (red) in an aged subject (Popa-Wagner, unpublished data).

♦ Recent work suggests that perfusion deficits in the elderly can trigger microglial activation and subsequent neuroinflammation, and shifts the CNS into a proinflammatory state, ultimately resulting in demyelination and neurodegeneration (**Figure 1**).
